# Effects of Shizhifang on NLRP3 Inflammasome Activation and Renal Tubular Injury in Hyperuricemic Rats

**DOI:** 10.1155/2017/7674240

**Published:** 2017-11-12

**Authors:** Yansheng Wu, Fei He, Yingqiao Li, Huiling Wang, Liqiang Shi, Qiang Wan, Jiaoying Ou, Xiaoying Zhang, Di Huang, Lin Xu, Pinglan Lin, Guanghui Yang, Liqun He, Jiandong Gao

**Affiliations:** ^1^Department of Nephrology, Shuguang Hospital Affiliated to Shanghai University of Traditional Chinese Medicine, TCM Institute of Kidney Disease of Shanghai University of Traditional Chinese Medicine, Shanghai Key Laboratory of Traditional Chinese Clinical Medicine, No. 528 Zhangheng Road, Shanghai 201203, China; ^2^Department of Nephrology, Xiamen Hospital of Traditional Chinese Medicine, No. 1739 Xianyue Road, Xiamen 361009, China; ^3^Department of Nephrology, Traditional Chinese Medicine Hospital of Langfang City, No. 108 North Yinhe Road, Langfang 065000, China; ^4^Department of Internal Medicine, Shanghai TCM-Integrated Hospital Affiliated to Shanghai University of Traditional Chinese Medicine, No. 184 Baoding Road, Shanghai 200082, China; ^5^Department of Laboratory, Shuguang Hospital Affiliated to Shanghai University of Traditional Chinese Medicine, No. 528 Zhangheng Road, Shanghai 201203, China; ^6^Department of Rheumatology and Immunology, Shuguang Hospital Affiliated to Shanghai University of Traditional Chinese Medicine, No. 528 Zhangheng Road, Shanghai 201203, China

## Abstract

**Objective:**

Uric acid (UA) activates the NLRP3-ASC-caspase-1 axis and triggers cascade inflammatory that leads to hyperuricemic nephropathy and hyperuricemia-induced renal tubular injury. The original study aims to verify the positive effects of the traditional Chinese medicinal formula Shizhifang (SZF) on ameliorating the hyperuricemia, tubular injury, and inflammasome infiltration in the kidneys of hyperuricemic lab rats.

**Method:**

Twenty-eight male Sprague-Dawley rats were divided into four groups: control group, oxonic acid potassium (OA) model group, OA + SZF group, and OA + Allopurinol group. We evaluated the mediating effects of SZF on renal mitochondrial reactive oxygen species (ROS) and oxidative stress (OS) products, protein expression of NLRP3-ASC-caspase-1 axis, and downstream inflammatory factors IL-1*β* and IL-18 after 7 weeks of animals feeding.

**Result:**

SZF alleviated OA-induced hyperuricemia and inhibited OS in hyperuricemic rats (*P* < 0.05). SZF effectively suppressed the expression of gene and protein of the NLRP3-ASC-caspase-1 axis through accommodating the ROS-TXNIP pathway (*P* < 0.05).

**Conclusion:**

Our data suggest that SZF alleviates renal tubular injury and inflammation infiltration by inhibiting NLRP3 inflammasome activation triggered by mitochondrial ROS in the kidneys of hyperuricemic lab rats.

## 1. Introduction

Economic development has led to marked changes in eating habits. The increased intake of fat, salt, and calories has elevated the prevalence of hyperuricemia and hyperuricemic nephropathy, leading to an increase in preventative research in the field of kidney health. Extensive epidemiological surveys and experimental research outcomes have revealed that hyperuricemia is closely associated with gout [1], diabetes [2, 3], metabolic syndrome [4], chronic heart failure [5], and nervous system disease [6, 7]. A growing body of evidence suggests that slightly elevated uric acid (UA) could lead to hypertension, retinal vascular disease, and renal injury. In Asian countries, 26.1% of men and 17.1% of women exhibit slightly elevated UA [8]. One survey [9] found that 120 million people in China have received diagnoses of hyperuricemia and that the age of onset is decreasing, with regions such as Shandong province achieving a hyperuricemia morbidity rate of 13.19%. The results of a recent cross-sectional study [10] validated an independent correlation between hyperuricemia and a reduced glomerular filtration rate in patients with CKD. Another study [11] showed that the prevalence of hyperuricemia in patients diagnosed with Stage 1–3 CKD is between 40% and 60% and that in patients with Stage 4 or 5 CKD is 70%. Therefore, UA levels have become a predictive factor for evaluating CKD development and subsequent renal inflammation has become a key factor in CKD progressing to end-stage renal disease (ESRD). Hence, the research and prevention of hyperuricemia have become global endeavours.

UA plays a proinflammatory role in numerous signalling pathways. Recent studies [12, 13] have found that soluble UA and UA crystals activate the NLRP3 (NLR family pyrin domain containing 3) inflammasome, causing proinflammatory cytokines such as IL-1*β* to mature and trigger congenital immune defence against danger signals such as infection and metabolic disorder. The NLRP3 inflammasome is a type of polyprotein complex formed through the interconnection and oligomerisation of NLRP3 receptor proteins, apoptosis-associated speck-like proteins containing a caspase recruitment domain (ASC), and caspase-1. NLRP3 receptor proteins can identify pathogen-associated molecular patterns and damage-associated molecular patterns (DAMPs). DAMPs are found in various components of the body, such as damaged tissues and substances released after tissue necrosis (e.g., adenosine triphosphate [ATP] or UA) [14]. Currently, NLRP3 receptor proteins are believed to be activated by the efflux of potassium ions, generated reactive oxygen species (ROS), or controlled by tissue protease released by ruptured lysosomes. Among these mechanisms, the most widely accepted for activation of NLRP3 is mitochondrial ROS generation [15]. Zhou et al. [16] observed an unexpected pivotal function in mitochondria during ROS generation. Specifically, the NLRP3 inflammasome is excited by ROS secreted by damaged mitochondria. These findings suggest that mitochondria are essential for both apoptosis and inflammatory responses. The release of ROS causes thioredoxin interacting proteins (TXNIP) to separate from oxidised thioredoxin-1 (Trx-1) proteins and bond with the leucine-rich repeat domain of NLRP3 receptor proteins, which consequently activates the NLRP3 inflammasome [17]. Therefore, the ROS-TXNIP pathway becomes a precondition for activation of the NLRP3-ASC-caspase-1 inflammatory pathway. Recent research [18] has shown that once the NLRP3-ASC-caspase-1 axis is activated, the inflammatory response mediated by caspase-1 triggers a type of programmed cell death: pyroptosis. Pyroptosis may be a newly identified mechanism for UA-induced renal inflammation and injury.

SZF is a traditional Chinese medicinal formula comprising plantago seeds, white mustard seeds, vaccaria seeds, and abutilon seeds. This formula, patented in China, has been used for over 20 years in the clinical treatment of hyperuricemia and hyperuricemic nephropathy, and the results have been reliable [19, 20]. To empirically elucidate the mechanisms underlying the effects of SZF on renal function, we used SZF to treat rats with mild hyperuricemia and then examined the interference of SZF on the NLRP3 signalling pathways in their kidneys. Based on this intervention, we hypothesised that SZF suppresses the renal ROS generation of the hyperuricemic lab rats, thereby inhibiting renal and oxidative stress (OS), obstructing the assembly of the NLRP3 inflammasome and activation of the NLRP3-ASC-caspase-1 axis, and reducing tubular inflammatory responses. Moreover, SZF may reduce renal cell pyroptosis and protect tubular cell function.

## 2. Materials and Methods

### 2.1. Animals

Twenty-eight healthy adult male Sprague-Dawley rats (SPF-grade) weighing 200 ± 20 g were examined. The rats were provided by the Shanghai Laboratory Animal Centre (SLAC). This study was conducted in accordance with safe animal testing specifications (Safety Certificate Number: SYXK-HU-2008-0016, Animal Ethics Code SZY201610008). The rats were kept at the Shanghai University of Traditional Chinese Medicine Animal Testing Centre at 25°C (relative humidity, 45%) with a 12-hour day/night cycle under artificial light. They were given ad libitum access to water. After an adaptation period of 1 week, we commenced with the proposed diet and interventional treatment. All animal tests were approved by the Shanghai University of Traditional Chinese Medicine Animal Testing Centre.

### 2.2. Reagents and Drugs

Oxonic acid potassium salt (OA, 156124) was purchased from Sigma-Aldrich (United States,) and standard feed containing 2% OA was prepared by the SLAC. SZF dry powder made by Shanghai Chinese Traditional Pharmaceutical Technology Co., Ltd. (China), was dissolved in distilled water to produce the equivalent of the crude drug concentration of 2.68 g/kg and was administered intragastrically. Allopurinol tablets (0.1 g, 121203) were purchased from Beijing Yanjing Pharmaceutical Co., Ltd. The UA testing kit (C012-1), creatinine testing kit (C011-2), urea nitrogen testing kit (C013-2), mitochondrial sampling and testing kit (G006), glutathione peroxidase testing kit (GSH-PX A005), MDA testing kit (A003-1), superoxide dismutase (SOD) testing kit (A001-3), and CAT testing kit (A007-1) were provided by Nanjing Jiancheng Bioengineering Institute (Nanjing, China). The enzyme-linked immunosorbent assay (ELISA) for rat ROS (DRE901Ra) was provided by AMEKO (China). The rat IL-18 (SU-B30650) and IL-1*β* (SU-B30419) ELISA kit were purchased from Kenuodi (Quanzhou, China) Biological Technology Co., Ltd. Rabbit anti-rat IL-18 (H-173) and ASC (N-15-R) antibodies were purchased from Santa Cruz Biotechnology (United States). Rabbit anti-rat 3-NT (APG8A) antibodies were purchased from Biovision (United States). Rabbit anti-rat IgG IL-1*β* (ab9787), caspase-1 (ab10862), TXNIP (ab86983), and 4-HNE (ab46545) were purchased from Abcam (British). Rabbit anti-rat IgG NLRP3 (NALP3; Nbp2-12446) was purchased from NOVUS (United States). The ready-to-use rabbit IgG-SABC immunohistochemical and SABC-AP (mice IgG) testing kits and DAB colour development kit were purchased from Boster (Wuhan, China). GAPDH antibodies (5174S) were purchased from Cell Signalling Technology (United States). The HRP secondary antibodies were provided by Jackson ImmunoResearch Laboratories. The mRNA sampling and testing kit was purchased from Tiangen Biotech (Beijing, China) Co., Ltd. The reverse transcription kit (RR036A) and SYBR Premix Ex Taq TM testing kit (RR420A) were provided by Takara Biotechnology Co., Ltd. (Japan).

### 2.3. SZF Fingerprint Identification

**Figure 1:**
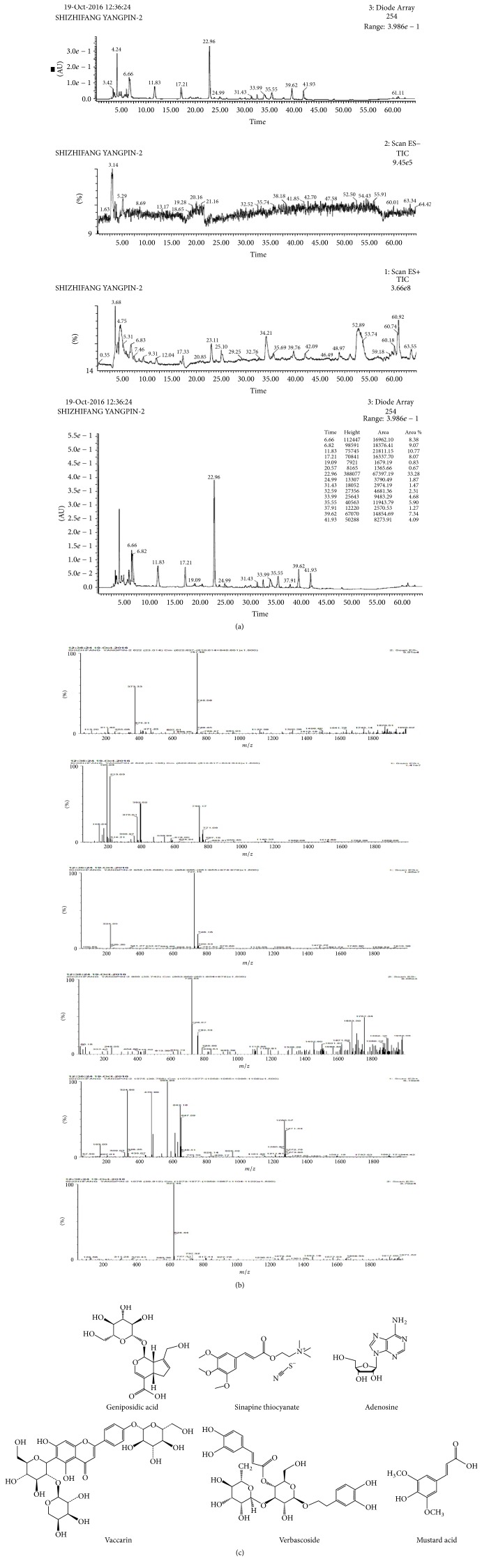
Results of SZF (Shizhifang) fingerprint identification. (a) Total ion chromatography of SZF. (b) Ion flow diagram corresponding with primary ingredients of geniposidic acid, calycosin-7-glucoside, vaccaria-glucoside, and sinapine thiocyanate, obtained through LC–MS analysis. (c) Main composition of SZF.

Shanghai Chinese Traditional Pharmaceutical Technology Co., Ltd. (China), was commissioned to perform fingerprint identification on the SZF. Vaccaria tablets (30 g; batch number 140220), white mustard seed tablets (30 g; batch number LY1505016), abutilon seed tablets (30 g; batch number 160517HY), and plantago seed tablets (60 g; batch number 151225) were obtained from Shanghai Union Dispensary Co. (China). The origins of the medicinal herbs used to produce these tablets were, respectively, Jilin, Anhui, Jiangxi, and Anhui provinces, China. The tablets were blended, crushed, and placed in herb bags according to the SZF formula. Each bag (one unit) was submerged in eight units of water for 1 hour and then heated under reflux twice for 1 hour each. The filtrates were combined and blended to produce the test solution. The test solution was analysed against several control samples (i.e., geniposidic acid, calycosin-7-glucoside, vaccaria-glucoside, and sinapine thiocyanate) and a blank (water) to produce the liquid fingerprints of the test solution. Water extraction was performed on the four medicinal herbs (i.e., fried plantago seeds, fried vaccaria, white mustard seeds, and abutilon seeds). A liquid analysis was then conducted on the extracts. Each extract was sampled six times to calculate the relative retention time and relative standard deviation of the relative peak area. The results showed that all extracts met the test requirements. Fingerprint similarity calculation software was employed to measure the extracts. The similarity of all six extracts was 100%, showing that the precision of the proposed method met the test requirements. We also observed the injection times (0, 2, 4, 6, 8, and 24 h) of the six extracts. The results indicated that all samples maintained excellent stability within 24 h. Fingerprint similarity calculations showed that the similarity of the six extracts was 99.9%, verifying that the reproducibility of the proposed method met the test requirements (Figures 1(a)–1(c)).

### 2.4. Establishing the Hyperuricemic Rat Model and Dosage

The 28 male rats were randomly divided into four groups which were named Control group, OA group, OA + Allopurinol group, and OA + SZF group based on weight. Each group contained seven rats. From Day 1, the rats in Control group were fed on a standard diet and the rest of rats were fed on a diet containing 2% OA to produce hyperuricemic model. All rats were given ad libitum access to water, and their diet was maintained for 7 weeks. The two treatment groups underwent their respective interventions from Day 1. The dosage for the SZF was based on the standards proposed by the Administration of Substances to Laboratory Animals (10 times the adult dosage per kilogram body weight). Allopurinol was dissolved in the drinking water of the OA + Allopurinol group (concentration, 150 mg/L). The control and OA groups were given equivalent amounts of distilled water. The intragastric volume was controlled at 2 ml/d for 7 weeks.

### 2.5. Sample Collection

The body weights and serum uric acid (Sua) levels of the rats were recorded before and after OA induction and at 2-week intervals. Blood samples were collected from the tail vein, left to clot in preiced tubes, and then centrifuged at 3000*g* at 4°C for 5 min to obtain the serum. Urine samples were collected with metabolic cages at 2-week intervals during drug treatment and centrifuged at 3000*g* for 5 min to remove impurities. The samples were stored at −20°C for the assays. Serum and urine UA, serum creatinine, and blood urea nitrogen levels were detected using specific commercial kits purchased from Nanjing Jiancheng Bioengineering Institute (Nanjing, China). Twenty-four-hour urine uric acid (Uua) was calculated using the following formula: Uua = urine UA concentration (*μ*mol/L) × urine volume (L) in 24 h. At the end of the drug intervention, all animals were deprived of food but not water until the following morning. Rats were anaesthetised with pentobarbital sodium (30 mg/kg i.p.) and blood samples were collected through the aorta abdominalis. The kidney was quickly dissected. Partial cortex tissues from the left kidney were immediately frozen in liquid nitrogen. Total cellular protein and RNA were extracted from kidney tissues. These samples were stored at −80°C until they were assayed. The separated serum and other partial left kidney tissues were stored at −80°C for measurement of the MDA, GSH-PX, SOD, CAT, and ROS levels.

### 2.6. Histopathology

The right kidney tissue was fixed for 1 day at room temperature in 90% ethanol. Renal biopsies were dehydrated with a graded series of alcohol and embedded in paraffin. Specimens were cut into 3-*μ*m thick sections on a rotary microtome and mounted on APES-coated glass slides. Each section was deparaffinised in xylene, rehydrated in decreasing concentrations of alcohol in water, and stained with HE, PAS, and Masson reagents sequentially.

### 2.7. Mitochondrial Extraction and OS Analysis

ROS were detected in the serum and kidney tissues by using an ELISA kit according to the manufacturer's protocol. The mitochondria from the cortex tissues were then extracted using a mitochondrial isolation kit (Nanjing Jiancheng Bioengineering Institute, Nanjing, China) according to the manufacturer's protocol. SOD, CAT, GSH-PX activity, and MDA levels in the kidney cortex tissue samples were measured using commercially available kits according to the manufacturer's instructions.

### 2.8. Immunohistochemistry

Rat kidney cortex tissues were fixed with 90% ethanol, embedded in paraffin, and sectioned transversely. Paraffin-embedded samples were deparaffinised prior to incubation with primary antibodies at 4°C overnight. HRP-conjugated secondary antibodies were incubated for 60 min at room temperature. Sections were examined using a microscope (Eclipse 80i, Nikon, Japan). The antibodies used for immunohistochemistry were anti-3-NT (1 : 100), anti-4HNE (1 : 100), anti-NLRP3 (1 : 50), anti-ASC (1 : 200), and anti-caspase-1 (1 : 100).

### 2.9. Real-Time PCR Analysis

Total RNA was isolated from individual rat kidneys with commercial kits for evaluating mRNA expression of NLRP3 inflammasome axis-related genes (rTXNIP, rNLRP3, rASC, rCaspase-1, rIL-1*β*, rIL-18) and glyceraldehyde 3-phosphate dehydrogenase (rGAPDH). Reverse-transcribed cDNA was obtained using the SuperScript First-Strand Synthesis kit (Takara). The primers used are summarised in Table 1. All primer sequences were checked in GenBank to avoid inadvertent sequence homologies. They were designed and synthesised by Sangon Biotechnology (Shanghai, China). Reactions were performed using SYBR Green PCR master mix (Applied Biosystems) in a BioRadiCycleriQ Detection System. As an internal control, rGAPDH levels were quantified in parallel with the target genes. Normalisation and fold changes for each gene were calculated using the 2-DeltaDelta C(T) method.

### 2.10. Western Blot

Approximately 100 mg of frozen rat kidney tissues was homogenised in 1 ml of RIPA buffer and then centrifuged at 10,000*g* for 20 min. The protein concentrations of the supernatants were measured according to the Bradford method. Total proteins were denaturalized in boiling water for 5 min. Equal amounts of total protein were separated onto 6%–12% SDS-PAGE and electrophoretically transferred to a polyvinylidenedifluoride (PVDF) membrane (Millipore, Shanghai, China) which was preactivated with methanol in the transferring buffer. Membranes were blocked with 5% skim milk for 2 h and incubated overnight with specific primary antibodies at 4°C. Immunoreactive bands were detected using HRP-conjugated goat anti-rabbit IgG as the secondary antibody (1 : 5000) (Jackson, Shanghai, China). Immunoreactive bands were visualised using a phototope-horseradish peroxidase Western blotting detection system (Cell Signaling Technologies, Beverly, MA) and quantified through densitometry with Molecular Analyst (Bio-Rad Laboratories, Hercules, CA). Primary antibodies included rabbit polyclonal antibodies against rTXNIP, rNLRP3, rASC, rCaspase-1, rpro-IL-1*β*, rIL-1*β*, and pro-IL-18, and rIL-18 (all 1 : 1000).

### 2.11. Measurement of Serum IL-1*β* and IL-18 Levels

IL-1*β* and IL-18 levels in serum were determined by the ELISA kits following the manufacturer's instruction.

### 2.12. Statistical Analysis

Data are expressed as the mean ± standard error of the mean or standard deviation (SD). Statistical analysis was performed by a one-way analysis of variance (ANOVA) followed by the Student-Newman-Keuls test. Differences were considered significant at *P* < 0.05. The figures were obtained using SPSS version 18.0.

## 3. Results

### 3.1. SZF Reduces Oxonic Acid Potassium-Induced Serum Uric Acid in Hyperuricemic Rats, Ameliorates Renal Inflammation and Injury, and Inhibits Collagen Proliferation

**Figure 2:**
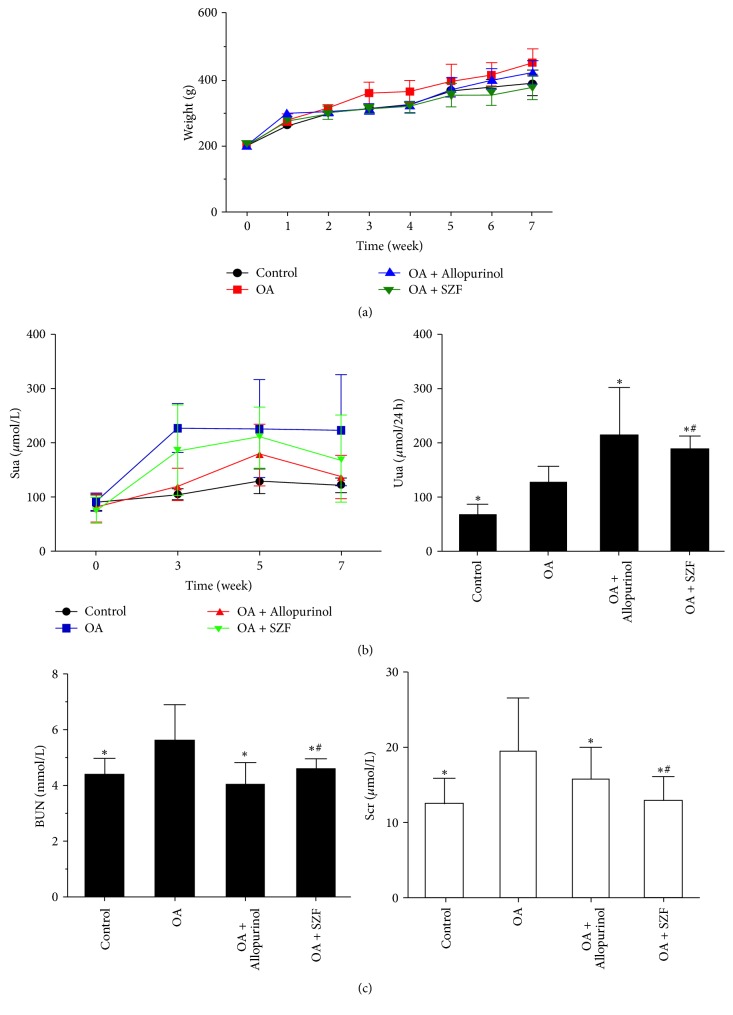
SZF improved the kidney function of rats with OA-induced hyperuricemia. (a) Changes in animal weight over the 7 weeks of animal modelling and treatment; OA: oxonic acid potassium salt; (b) changes in Sua in the tail vein over the 7 weeks of animal modelling and treatment and the 24 h urinary UA excretion conditions at the end of the 7 weeks; UA: uric acid; Sua: serum uric acid; Uua: 24 h urinary uric acid excretion; (c) BUN and Scr levels in the abdominal aorta of the various groups at the end of the 7 weeks of animal modelling and treatment. BUN: blood urea nitrogen; Scr: serum creatinine. Control: the rats received standard diet and no intervention; OA: the rats received 2% OA diet to produce hyperuricemic model; OA + Allopurinol: the rats received 2% OA diet and drinking water dissolved in allopurinol; OA + SZF: the rats received 2% OA diet and Traditional Chinese Medicine formula of Shizhifang intragastrically. Data are expressed as the mean ± SD (*n* = 7). ^*∗*^*P* < 0.05 versus the OA group. ^#^*P* > 0.05 versus the OA + Allopurinol group.

In clinical settings, serum uric acid in hyperuricemic patients is approximately 1.5 to 2 times higher than that in healthy individuals. Hyperuricemia typically develops into a chronic, progressive condition. Therefore, we used an animal model of mild hyperuricemia resembling clinical characteristics by mixing 2% oxonic acid potassium salt (OA) into the diet of male Sprague-Dawley rats to induce nonurate deposition [21]. The rats in the treatment groups were generally in good condition, and none of them died during the experimental process. The changes in animal weight are illustrated in Figure 2(a). Observation of the control, OA model, and treatment groups indicated significant differences (*P* < 0.05). The Sua levels of the lab rats at Weeks 0, 3, 5, and 7 as well as their 24 h urinary UA excretion measurements are illustrated in Tables 2 and 3 and Figure 2(b). At the end of Week 7, the Sua levels of the rats in the model group were approximately 1.8 times higher than those in the control group (OA group: 223.3 ± 102.41 *μ*mol/L; controls: 121.38 ± 12.66 *μ*mol/L), which was consistent with the clinical characteristics of mild elevation of serum uric acid. A comparison between the two treatment groups showed no significant statistical differences (*P* > 0.05), suggesting that SZF and allopurinol achieved similar effects in reducing Sua. The total 24 h urinary UA excretion volume of the model group was significantly higher than that of the control group (*P* < 0.05), and those of the SZF and allopurinol groups were significantly higher than that of the OA group (*P* < 0.05). These observations suggest that the SZF effectively reduced Sua by promoting UA excretion.

Allopurinol is a widely recognised xanthine oxidase (XOD) inhibitor. It suppresses XOD activity and disrupts UA production, thereby reducing the effects of UA. In this study, the urinary UA excretion volume of the allopurinol group far exceeded that of the control and OA groups, verifying the effects of allopurinol on UA excretion. These observations are consistent with those reported in a previous study [22]. The serum creatinine and blood urea nitrogen levels of the various groups at the end of Week 7 are shown in Figure 2(c) and Table 4. Compared with the controls, the BUN and Scr levels of OA group were significantly higher (*P* < 0.05), verifying that elevated UA damaged kidney function. However, the BUN and Scr levels of the lab rats receiving the SZF and allopurinol treatments decreased.

**Figure 3:**
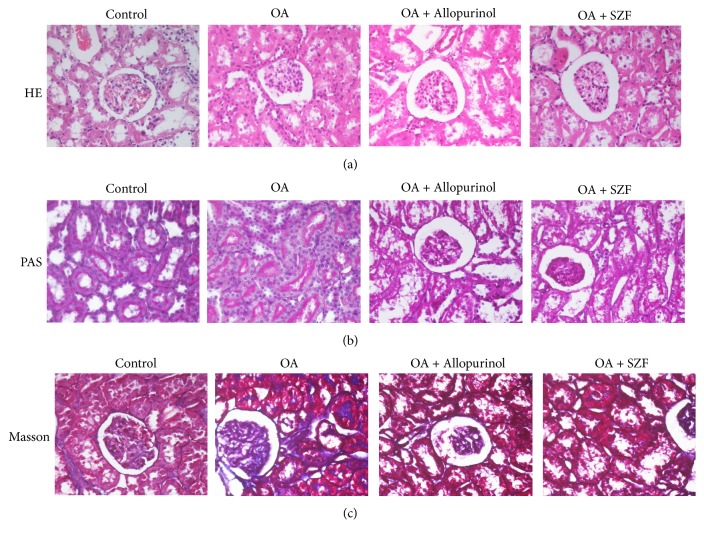
SZF improved renal pathological morphology and reduced cell damage in the hyperuricemic rats. (a) H&E staining results of the renal tissue (magnification 400x); (b) PAS staining results of the renal tissue (magnification 400x); (c) Masson's staining results of the renal tissue (magnification 400x).

Haematoxylin and eosin (H&E) staining results showed that the kidney tissue structures of the controls were relatively normal (Figure 3(a)). Local swelling in the renal proximal tubular epithelial cells and significant increases in vacuolar degeneration and inflammatory cell infiltration were observed in the model group. In addition, a small portion of tubular epithelial cells exhibited an increase in epithelial cell nuclei shedding due to vacuolar degeneration. No differences were observed in the glomeruli. For the OA + SZF and OA + Allopurinol groups, there was a similar lack of changes in the glomeruli. These groups exhibited reduced swelling in the renal proximal tubular epithelial cells, vacuolar degeneration, and inflammatory cell infiltration. According to the Periodic Acid-Schiff (PAS) and Masson's trichrome staining results, the controls showed orderly renal tubule arrangements, uniform cell walls, normal brush-border microvilli formation on the inner surface of the tubular lumens, basement folds formed from basal cell invagination, and no renal interstitial fibrosis (Figures 3(b) and 3(c)). The OA group showed disorderly renal tubule arrangements, inflamed tubules and interstitial cell infiltration, narrow lumens, changes in the brush-border microvilli structures of the tubular walls, partial shedding, incoherent basal membranes, and fibrosis of the tubular lumens and interstitia. The OA + Allopurinol group showed orderly renal tubule arrangement, slight tubular inflammation and interstitial cell infiltration, expanded lumens, vacuolar degeneration, changes in the brush-border microvilli structures of the tubular walls, incoherent basal membranes, and slight fibrosis in the tubular lumens and interstitia. The OA + SZF group showed orderly renal tubule arrangements, uniform and consistent cell walls, rough luminal surfaces, no evident changes in microvilli structures, basal cell invagination forming basement folds, and no evident fibrosis in the tubular lumens and interstitia.

### 3.2. SZF Inhibits OS in Hyperuricemic Lab Rats by Suppressing Mitochondrial ROS

**Figure 4:**
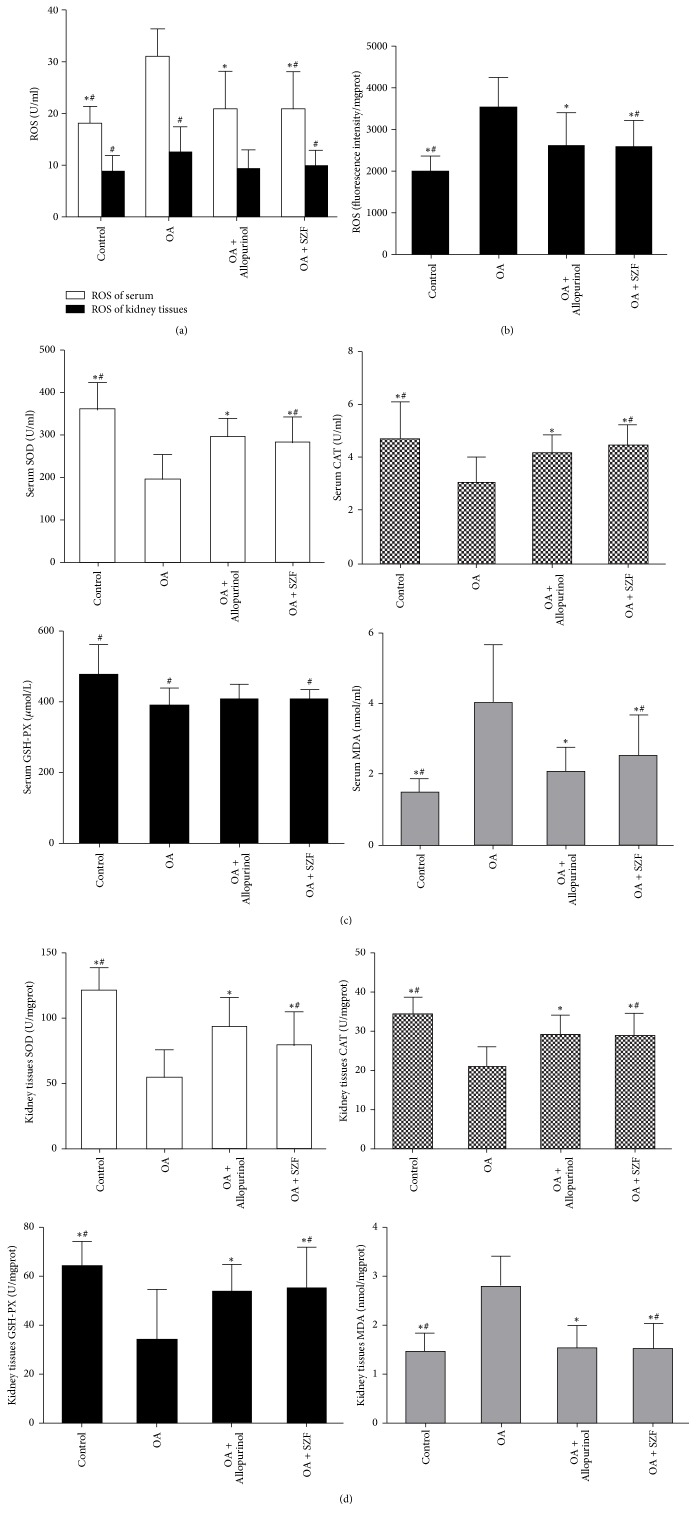
SZF inhibited the OS of hyperuricemic rats by suppressing mitochondrial ROS. (a) ROS levels in the serum and renal cortex tissue; ROS: reactive oxygen species; OS: oxidative stress; (b) relative fluorescence intensity of the mitochondrial ROS after the mitochondria were isolated from the tissue cells of the renal cortex; (c) SOD, CAT, GSH-PX, and MDA levels in the serum of the various groups; SOD: superoxide dismutase; CAT: catalase; GSH-PX: glutathione peroxidase; MDA: malondialdehyde; (d) SOD, CAT, GSH-PX, and MDA levels in the renal tissue of the various groups. Data are expressed as the mean ± SD (*n* = 7). ^*∗*^*P* < 0.05 versus the OA group. ^#^*P* > 0.05 versus the OA + Allopurinol group.

**Figure 5:**
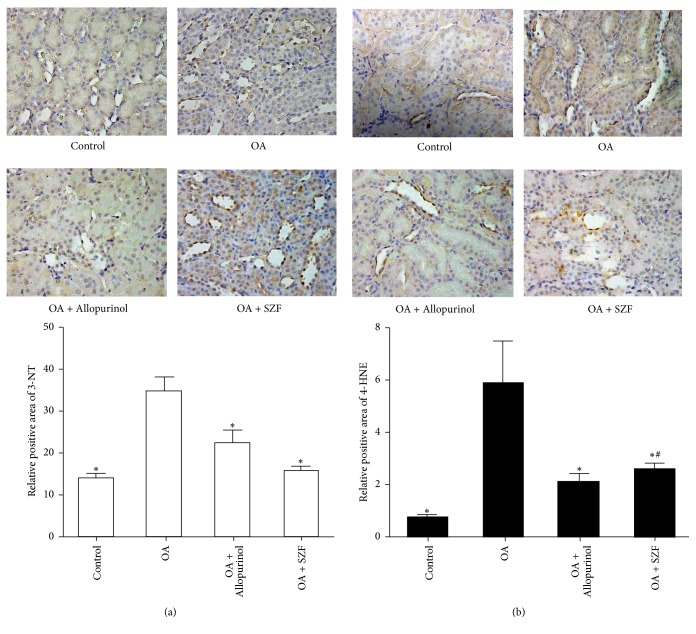
SZF affected the expression of the OS products 3-NT and 4-HNE in renal tissue with high UA. (a) 3-NT immunohistochemical staining (magnification 200x) and positive area results in the renal tissue; 3-NT: 3-nitrotyrosine; (b) 4-HNE immunohistochemical staining (magnification 400x) and positive area results in the renal tissue; 4-HNE: 4-hydroxy aldehyde. Data are expressed as the mean ± SD (*n* = 7). ^*∗*^*P* < 0.05 versus the OA group. ^#^*P* > 0.05 versus the OA + Allopurinol group.

Physiologically, ROS serve as secondary messengers for cell signals and protein modifications. They control cell growth and proliferation and regulate the transcription and activity of different molecules. Excess release of ROS may trigger renal tissue and body OS, change redox states, permanently damage large molecules (e.g., DNA, RNA, proteins, and lipids), and interfere with crucial signalling pathways for redox reactions [23]. In this study, we tested the ROS expression in the serum and renal tissue of rats with OA-induced hyperuricemia (Figure 4(a)). As anticipated, serum collected from the rats in the OA group showed elevated ROS levels (*P* < 0.05; statistically significant) and renal tissue (*P* > 0.05; statistically nonsignificant). By comparison, the treatment groups showed similar effects in suppressing ROS release. To identify the sources of renal ROS in hyperuricemic rats with UA-induced renal inflammation, we obtained mitochondria from renal tissue, performed mitochondria isolation, and tested the ROS levels in the mitochondria (Figure 4(b)). The results verified that mitochondrial ROS serves a key function in the renal tissue and in vivo OS. These observations are consistent with the findings of Ives et al. [24], who contended that mitochondrial ROS originating from XOD is the main source of ROS for kidney inflammasome activation. Furthermore, we tested the OS level of the hyperuricemic rats in their serum and renal tissue (Figures 4(c) and 4(d)) and found a significant imbalance between the superoxide dismutase (SOD), catalase (CAT), and glutathione peroxidase (GSH-PX) of the antioxidant systems and the malondialdehyde (MDA) of the peroxidation systems in the rats' bodies and specifically kidneys. Subsequently, SZF and allopurinol intervention restored the dynamic balance of these compounds to a certain extent. To elucidate the influence of OS on renal tissue inflammation and cell damage, we applied immunohistochemistry to test the contents of the tyrosine-specific nitration products (3-NT; Figure 5(a)) and lipid peroxide (4-HNE; Figure 5(b)) in various groups. The results indicated that 3-NT and 4-HNE were primarily expressed in the cytoplasm of tubular epithelial cells. The OA group exhibited elevated 3-NT and 4-HNE expression in the renal interstitia, suggesting that OS damages the kidneys and that SZF can reduce OS-induced renal inflammation and tubular injury. These results also suggest that high UA levels can prompt mitochondria to produce excess ROS, leading to an imbalance between renal and in vivo oxidation and antioxidation systems and thereby causing OS and an increased probability of renal damage. SZF can repair OS-induced injury. To elucidate the interaction between UA-stimulated ROS and renal inflammation, we examined the effects of UA on the pathways concerning renal anti-inflammation, NLRP3 inflammasome activation, and SZF intervention.

### 3.3. SZF Inhibits Activation of the NLRP3-ASC-Caspase-1 Axis by Suppressing TXNIP

**Figure 6:**
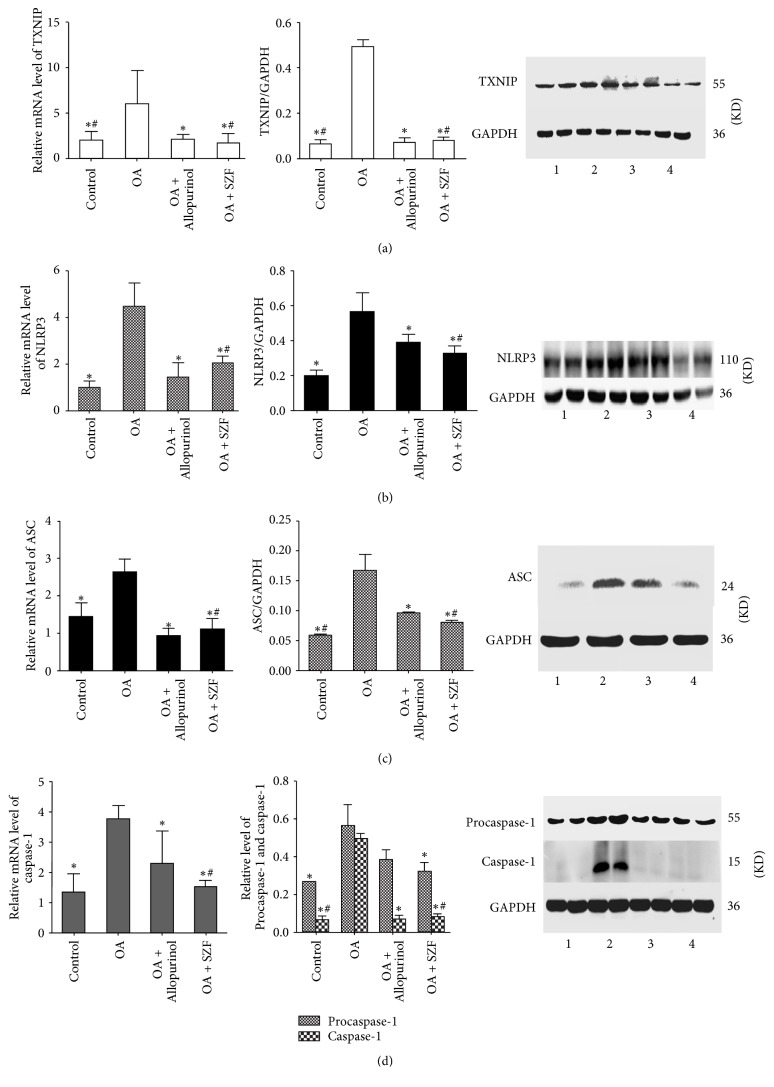
SZF inhibited the activation of the NLRP3-ASC-caspase-1 axis by suppressing TXNIP. Gene and protein expression levels in the renal tissue: (a) TXNIP; (b) NLRP3; (c) ASC; (d) caspase-1 and Procaspase-1. Protein levels were determined by Western blotting, were quantified through densitometry, and are expressed as the optical density ratio to GAPDH. mRNA levels were determined through real-time PCR. The numbers in the Western blot figures represent groups: 1 means Control, 2 means OA, 3 means OA + Allopurinol, and 4 means OA + SZF. Data are expressed as the mean ± SD (*n* = 3). ^*∗*^*P* < 0.05 versus the OA group. ^#^*P* > 0.05 versus the OA + Allopurinol group.

**Figure 7:**
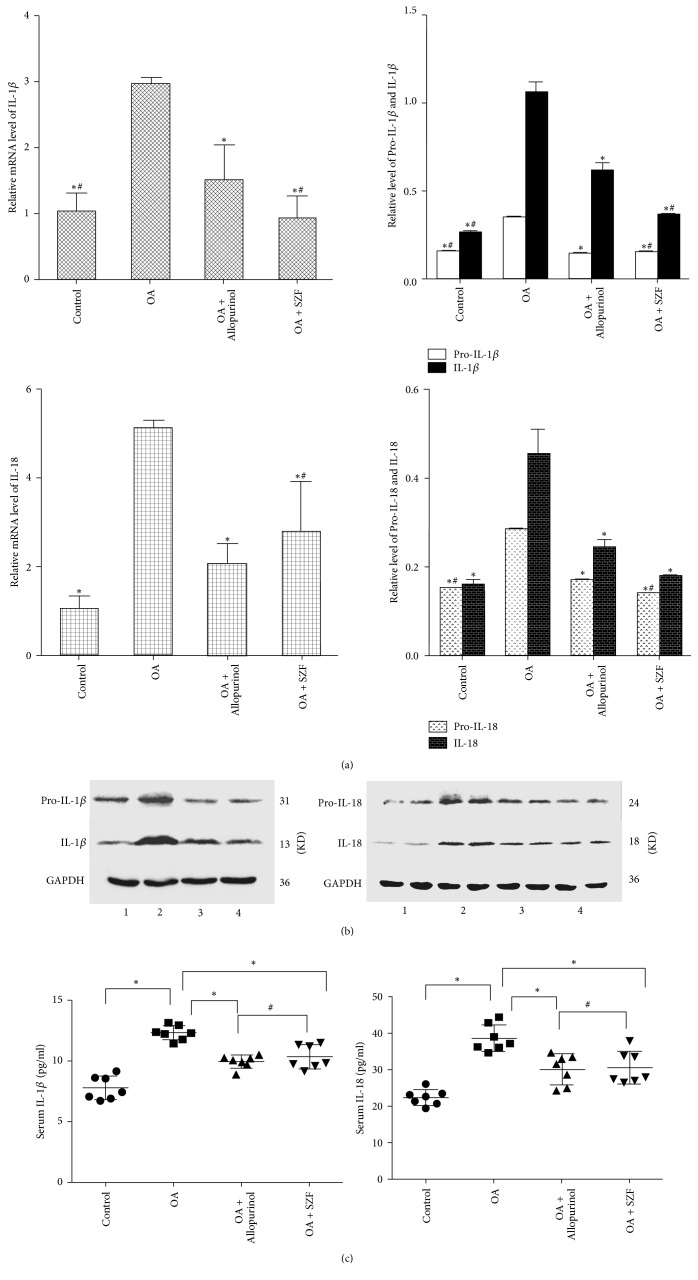
SZF reduced the levels of IL-1*β* and IL-18 in serum and kidney tissues of hyperuricemic rats. Gene and protein expression levels in the renal tissue: (a) The mRNA expression of IL-1*β* and IL-18 by Real-time PCR (*n* = 3); (b) The protein expression of IL-1*β* and IL-18 by Western blot (*n* = 3), The numbers in the Western blot figures represent groups, 1 means Control, 2 means OA, 3 means OA + Allopurinol, 4 means OA + SZF; (c) The serum inflammatory cytokines levels of IL-1*β* and IL-18 by ELISA (*n* = 7). Data are expressed as the mean ± SD. ^*∗*^*P* < 0.05 versus the OA group. ^#^*P* > 0.05 versus the OA + Allopurinol group.

**Figure 8:**
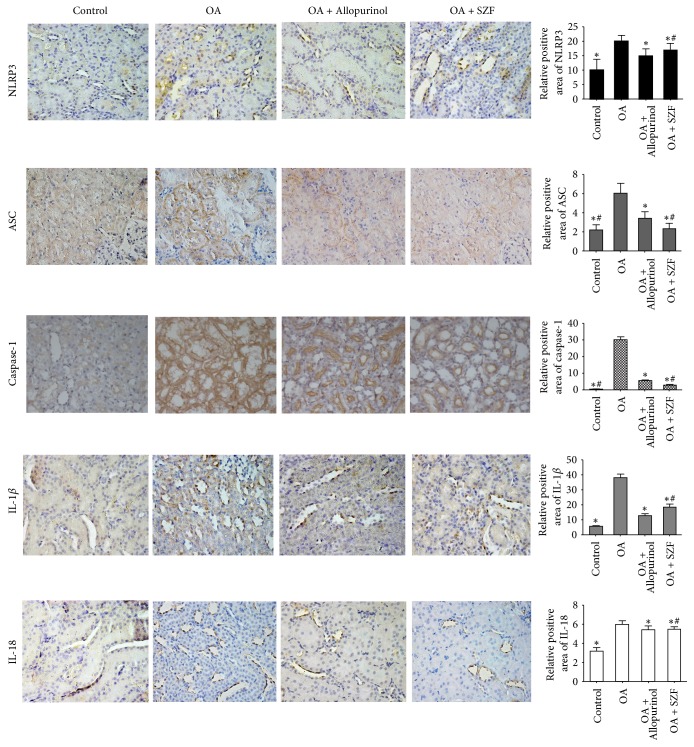
SZF affected the localization and expression of the NLRP3-ASC-caspase-1 axis proteins. NLRP3, ASC, caspase-1, IL-1*β*, and IL-18 immunohistochemical staining (magnification 200x) and positive area results in the renal tissue. Data are expressed as the mean ± SD (*n* = 7). ^*∗*^*P* < 0.05 versus the OA group. ^#^*P* > 0.05 versus the OA + Allopurinol group.

UA activates the inflammasome molecular complex NLRP3 in the cells through the ROS-TXNIP pathway, leading to the maturation and release of inflammatory factors IL-1*β* and IL-18. We performed real-time PCR and Western blotting to test the TXNIP mRNA and protein expression in the renal tissue of the hyperuricemic rats. Unsurprisingly, TXNIP mRNA and protein expression in the renal tissue of the rats in OA group were significantly higher than those in the other groups (*P* < 0.05). SZF and allopurinol effectively suppressed TXNIP mRNA and protein expression. SZF was particularly exceptional in suppressing TXNIP (Figure 6(a)). We then examined the influences of SZF on the activation index of the downstream NLRP3-ASC-caspase-1 axis in the TXNIP. First, we tested the mRNA and proteins in the NLRP3-ASC-caspase-1 axis (Figures 6(b)–6(d)) and found elevated mRNA and protein expression in the hyperuricemic rats (*P* < 0.05). SZF effectively suppressed the activation of the NLRP3-ASC-caspase-1 axis, indicating that the suppression effect was due to suppression of the ROS-TXNIP pathway (OS index). Second, we tested the mRNA and protein expression of the downstream inflammatory factors Pro-IL-18, IL-18, Pro-IL-1*β*, and IL-1*β* in the NLRP3-ASC-caspase-1 axis (Figures 7(a) and 7(b)). Moreover, we detected the IL-1*β* and IL-18 levels in serum by the ELISA kits (Figure 7(c)). Common results showed that SZF effectively reduced the inflammatory cytokines levels of IL-1*β* and IL-18 in serum and kidney tissues of hyperuricemic rats (*P* < 0.05). Immunohistochemical staining was adopted for a localization analysis (Figure 8). The results showed that NLRP3, ASC, caspase-1, IL-18, and IL-1*β* were all present in the tubular epithelial cytoplasm and were rarely expressed in the glomeruli. Examination of the real-time PCR, Western blotting, and immunohistochemistry results verified that SZF could effectively suppress the maturation and release of IL-18 and IL-1*β*, thus inhibiting renal inflammation. According to the results, UA stimulates excess ROS production and activates OS in the kidneys, which affects TXNIP activity and prompts TXNIP to bond with NLRP3, further triggering the assembly of the NLRP3 inflammasome. This process continues to activate the NLRP3-ASC-caspase-1 axis to release inflammatory factors and trigger renal inflammatory responses. SZF suppresses the activation of the NLRP3-ASC-caspase-1 axis by inhibiting TXNIP, thus ameliorating renal inflammation.

## 4. Discussion

An increasing amount of evidence suggests that UA-induced inflammatory responses are the central mechanisms of tubular injury in hyperuricemic rodents [25] and that the activation of NLRP3 and its mediating effects on inflammatory responses play key roles in various renal diseases [26]. Cristóbal-García et al. [27] found that OA-induced long-term hyperuricemia promoted OS in the renal cortex, damaged mitochondria, and reduced ATP levels. Park et al. [28] proposed the dynamics of mitochondrial damage, explaining that defective mitochondrial fission augments NLRP3 inflammasome activation and triggers abnormal inflammatory responses, confirming that NLRP3 activation and NLRP3-mediated inflammatory responses significantly influence inflammation in renal interstitia. The findings of numerous previous studies have verified that mitochondrial ROS activates the NLRP3 inflammasome through TXNIP, triggering a rapid renal inflammation response. As a sensitive signal complex regulator in cell redox, Trx/TXNIP has become a key point of connection between redox regulation and disease [29]. During cell quiescence, TXNIP and Trx-1 combine and remain deactivated. Trx-1 controls the ROS levels in cells and suppresses OS. When high levels of ROS are present in cells in a state of OS, TXNIP separates from Trx-1 and bonds with NLRP3 to activate the inflammasome and regulate inflammation signals [30, 31]. In this study, we observed high expression of mitochondrial ROS and TXNIP in the renal tissue under elevated UA conditions, which triggered NLRP3 overexpression and renal inflammation. However, ROS was suppressed by the allopurinol and SZF interventions, in turn suppressing OS and maintaining the TXNIP–Trx bond. This bond suppressed TXNIP activity and prevented it from bonding with NLRP3, thereby preventing the activation of the inflammasome axis and ameliorating renal inflammation and injury.

UA promotes activation of downstream caspase-1 in the NLRP3 inflammasome, creating a key platform for the activation and release of proinflammatory factors. The formation of myoblast caused by inflammatory cell aggregation and infiltration and the activation of inflammatory and proinflammatory factors are essential contributors to renal fibrosis, under which CKD progresses to ESRD. Immune inflammation triggers injury repair responses, leading to fibrosis and scarring and causing substantial function degradation. Ryu et al. [32] fed Sprague-Dawley rats standard feed containing 2% OA for 6 weeks and found that UA promoted the epithelial-mesenchymal transition of tubular epithelial cells. Hyperuricemia-induced EMT occurred ahead of renal interstitial fibrosis. The reduction in E-cadherin expression and increase in *α*-SMA expression indicated UA-induced phenotype transformation in the tubular epithelial cells. The activation of Snail and Slug transcription factors reduced E-cadherin synthesis and increased degradation of E-cadherin by ubiquitin, which promoted the phenotype transformation in the tubular epithelial cells. Mazzali et al. [21] analysed lab rats with OA-induced hyperuricemia and verified that hyperuricemia triggered systematic hypertension and ischaemic renal damage. Collagen deposition, macrophage infiltration, and increased osteopontin expression were observed in the tubular and interstitial areas, suggesting that UA participates in renal inflammation and triggers tubular and interstitial fibrosis. Through PAS and Masson's trichrome staining, we verified that elevated UA levels could cause renal inflammation and interstitial fibrosis by activating the NLRP3 inflammasome. SZF suppressed this activation, thereby inhibiting renal interstitial inflammation and alleviating renal fibrosis.

NLRP3 controls the activation of caspase-1, which is a prerequisite for IL-1*β* and IL-18 maturation [33] and contributes to pyroptosis through canonical inflammasome pathway. Recently, Shi et al. [34] and Kayagaki et al. [35] have found that caspase-1 mediates the cleaving of gasdermin D (GSDMD) to form aminoterminal fragments that induce pyroptosis activity. The canonical inflammatory pathway caspase-1-GSDMD serves a decisive function in pyroptosis, triggering it when the inflammasome downstream molecule caspase-1 cleaves GSDMD [36]. Chen et al. [37] asserted that cadmium triggers the mediating effects of caspase-1 on the pyroptosis and inflammation responses of vascular endothelial cells. The staining results of caspase-1 activation and the positive SYTOX green verified that cadmium activates the NLRP3 inflammasome in endothelial cells to trigger pyroptosis and produce ROS. Yang et al. [38] observed a significant increase in caspase-1 and IL-1*β* proteins associated with pyroptosis in ischemia/reperfusion- (I/R-) induced rats and discovered pyroptosis pore formation in NRK-52E cells and lactate dehydrogenase release during hypoxia and reoxygenation, verifying the occurrence of epithelial cell pyroptosis. Chung et al. [39] developed a unilateral ureteral obstruction (UUO) animal model of rats with kidney damage. Analysis outcomes showed tubular and interstitial inflammation infiltration in the obstructive side of the kidney in addition to several pathological changes such as fibrosis, autophagy, apoptosis, and pyroptosis. The UUO model exhibited increased expression of the caspase-1 and IL-1*β* proteins associated with pyroptosis in the kidneys. The application of catechin therapy using Nrf-2 agonist effectively protected mitochondrial function, improved renal blood flow, and significantly reduced the OS, inflammation, fibrosis, and three types of programmed cell death in kidneys with UUO.

Although we did not perform an in vitro analysis to observe the regulating effects of the SZF on cell pyroptosis, we investigated the changes in the expression levels of caspase-1, an activation protein for canonical pyroptosis pathways, and the subsequent effects of the maturity and release of inflammatory factors, thus deepening our understanding of pyroptosis in renal tissue. Our results indicated that the SZF effectively suppressed the activation of the NLRP3-ASC-caspase-1 axis, reducing the expression of caspase-1 proteins and preventing them from cleaving GSDMD. This process obstructs pyroptosis pathways, protecting the integrity of the cell membrane and ensuring normal cell function.

## 5. Conclusion

In summary, we verified that, through the suppression of mitochondrial ROS in the kidneys of hyperuricemic lab rats, SZF inhibits NLRP3 inflammasome activation, thereby alleviating tubular inflammation, cell pyroptosis, and renal interstitial fibrosis. We found that SZF demonstrated excellent performance in reducing Sua, protecting kidney function, and ameliorating renal inflammation and fibrosis. Compared with allopurinol, a classic drug for treating high levels of UA, SZF does not induce allergic reactions and is not toxic to the liver or kidneys. We delineated the therapeutic properties of SZF and its value for subsequent research and clinical applications.

## Figures and Tables

**Table 1:** PCR primer sequence.

Gene name	Primer sequence (5′–3′)	*Tm* (°C)
TXNIP	F 5′ GGCTTTTCTCGATCGTCCCA 3′	60.11
	R 5′ AGAGGCAGAAAGCGTTGAGT 3′	59.61
NLRP3	F 5′ ACGGCAAGTTCGAAAAAGGC 3′	59.97
	R 5′ AGACCTCGGCAGAAGCTAGA 3′	60.03
ASC	F 5′ AGACATGGGCATACAGGAGC 3′	59.53
	R 5′ GCAATGAGTGCTTGCCTGTG 3′	60.39
Caspase-1	F 5′ AAGAAGGTGGCGCATTTCCT3′	60.25
	R 5′ GACGTGTACGAGTGGGTGTT3′	59.97
IL-1*β*	F 5′ AGGATTGCTTCCAAGCCCTTGACT 3′	64.44
	R 5′ ACAGCTTCTCCACAGCCACCATGA 3′	66.22
IL-18	F 5′ ACAAAAGAAACCCGCCTGTG 3′	59.26
	R 5′ TGTGTCCTGGCACACGTTTC 3′	61.09
GAPDH	F 5′ TGTGAACGGATTTGGCCGTA3′	59.96
	R 5′ GATGGTGATGGGTTTCCCGT 3′	60.03

**Table 2:** Serum uric acid levels in each group at different time points (*μ*mol/L, mean ± SD).

Group	*N*	Week 0	Week 3	Week 5	Week 7
Control	7	89.56 ± 14.18	104.35 ± 12.49	128.56 ± 23.96^*∗*^	121.38 ± 12.66^*∗*^
OA	7	91.69 ± 19.31	226.45 ± 46.26	224.8 ± 90.59	222.3 ± 102.41
OA + Allopurinol	7	81.47 ± 26.17	119.93 ± 32.24	179.01 ± 58.23^*∗*^	137.57 ± 42.71^*∗*#^
OA + SZF	7	72.95 ± 25.57	183.96 ± 82.93	210.65 ± 54.39	167.26 ± 82.94^*∗*^

^*∗*^ *P* < 0.05 versus the OA group. ^#^*P* > 0.05 versus the OA + Allopurinol group.

**Table 3:** Total urine uric acid excretion in each group (mean ± SD).

Group	*N*	Urine volume (L/24 h)	Uua (*μ*mol/L)	Uua (*μ*moL/24 h)
Control	7	0.036 ± 0.01	1969.34 ± 808.21	64.00 ± 20.78^**∗**^
OA	7	0.034 ± 0.01	4271.16 ± 2429.5	124.39 ± 29.39
OA + Allopurinol	7	0.051 ± 0.00	4102.0 ± 1576.09	211.91 ± 88.69^**∗**#^
OA + SZF	7	0.045 ± 0.02	5254.3 ± 3916.99	186.51 ± 24.72^**∗**^

^*∗*^ *P* < 0.05 versus the OA group. ^#^*P* > 0.05 versus the OA + Allopurinol group.

**Table 4:** Blood urea nitrogen and serum creatinine of rats in each group (mean ± SD).

Group	*N*	BUN (mmol/L)	Scr (*μ*mol/L)
Control	7	4.40 ± 0.56^*∗*^	12.45 ± 3.43^*∗*^
OA	7	5.58 ± 1.30	19.54 ± 6.94
OA + Allopurinol	7	3.99 ± 0.82^*∗*^	12.93 ± 3.15^*∗*^
OA + SZF	7	4.57 ± 0.40^*∗*#^	15.79 ± 4.17^#^

^**∗**^ *P* < 0.05 versus the OA group. ^#^*P* > 0.05 versus the OA + Allopurinol group.
